# Transcranial random noise stimulation and cognitive training to improve learning and cognition of the atypically developing brain: A pilot study

**DOI:** 10.1038/s41598-017-04649-x

**Published:** 2017-07-05

**Authors:** Chung Yen Looi, Jenny Lim, Francesco Sella, Simon Lolliot, Mihaela Duta, Alexander Alexandrovich Avramenko, Roi Cohen Kadosh

**Affiliations:** 1 Department of Experimental Psychology, University of Oxford, Oxford, OX1 3UD United Kingdom; 2 Fairley House School, London, SW1P 4AU UK; 3 Cavendish Laboratory, University of Cambridge, Cambridge, CB3 0HE UK

## Abstract

Learning disabilities that affect about 10% of human population are linked to atypical neurodevelopment, but predominantly treated by behavioural interventions. Behavioural interventions alone have shown little efficacy, indicating limited success in modulating neuroplasticity, especially in brains with neural atypicalities. Even in healthy adults, weeks of cognitive training alone led to inconsistent generalisable training gains, or “transfer effects” to non-trained materials. Meanwhile, transcranial random noise stimulation (tRNS), a painless and more direct neuromodulation method was shown to further promote cognitive training and transfer effects in healthy adults without harmful effects. It is unknown whether tRNS on the atypically developing brain might promote greater learning and transfer outcomes than training alone. Here, we show that tRNS over the bilateral dorsolateral prefrontal cortices (dlPFCs) improved learning and performance of children with mathematical learning disabilities (MLD) during arithmetic training compared to those who received sham (placebo) tRNS. Training gains correlated positively with improvement on a standardized mathematical diagnostic test, and this effect was strengthened by tRNS. These findings mirror those in healthy adults, and encourage replications using larger cohorts. Overall, this study offers insights into the concept of combining tRNS and cognitive training for improving learning and cognition of children with learning disabilities.

## Introduction

While 2 to 3 children in every classroom with learning disabilities^1^ are predicted to endure negative socioeconomic outcomes in adulthood^2^, few behavioural interventions are effective^3, 4^. Given the limited success of behavioural interventions in modulating plasticity of the atypically developing brain^5^, exploration of innovative approaches is timely.

Transcranial random noise stimulation (tRNS) is a form of transcranial electrical stimulation that involves the application of random noise oscillations above selected brain regions to modulate cortical plasticity^6^. Its application in adults has been considered safe and painless within recommended guidelines^7^. One of the proposed mechanisms of tRNS is the increase of neuronal excitability via stochastic resonance, whereby weak neural signal detection in the central nervous system is enhanced when ‘noise’ is added^8^. While tRNS during cognitive training has shown to improve learning of healthy adults with effects that lasted for months^9^, its practical implications in the real-world (i.e., training and transfer effects on everyday tasks in non-laboratory settings) remain unexplored. In addition, its potential for remediating neural atypicalities in developing brains, which are associated with a greater degree of neuroplasticity^10^ has been proposed^5^ and debated^11^, but not empirically tested.

**Figure 1:**
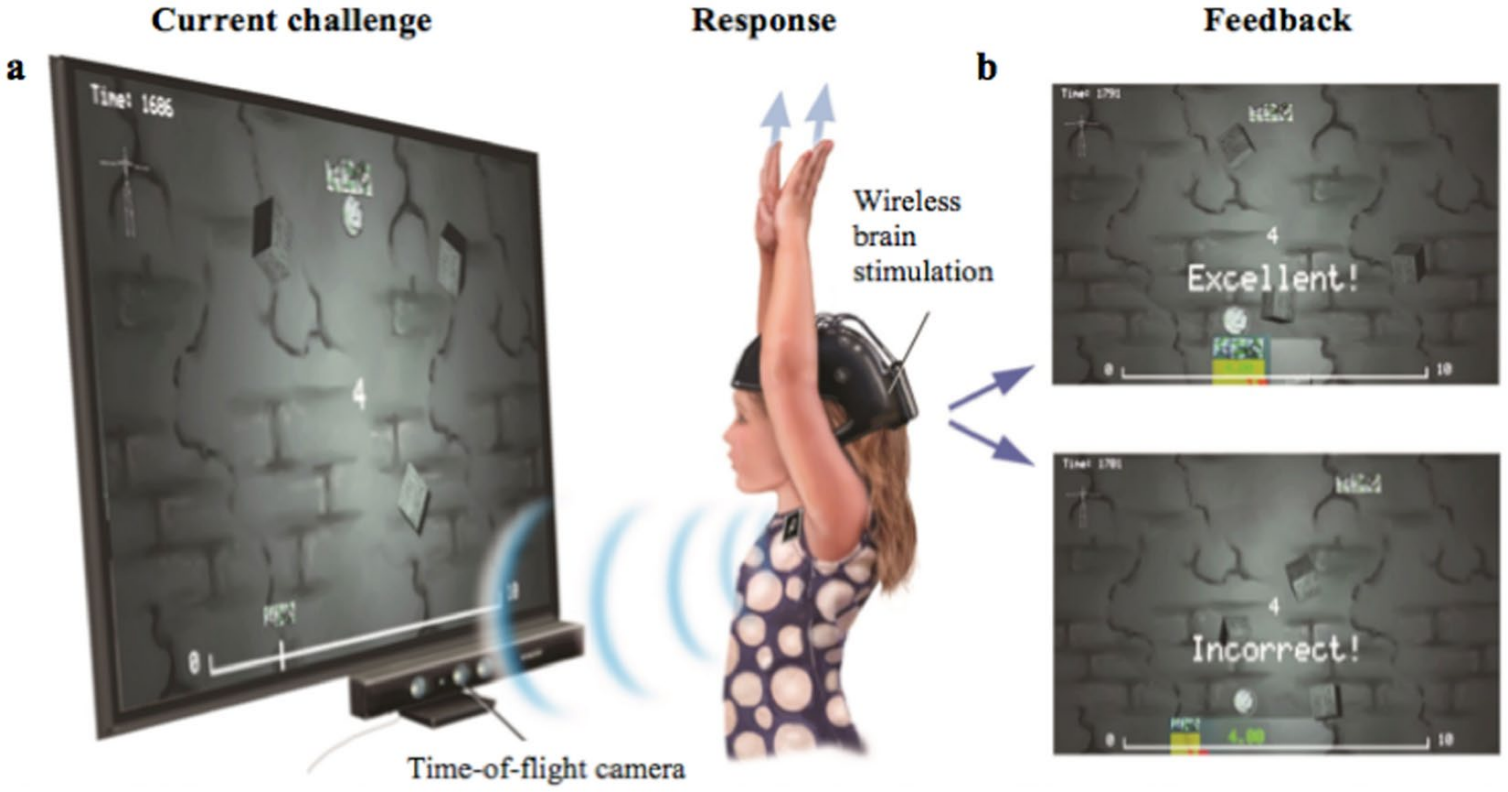
Transcranial random noise stimulation coupled with cognitive training to improve learning of children with mathematical learning disabilities at school (**a**) Illustration of a child moving from side-to-side to map a number on a number line while receiving transcranial random noise stimulation from a wireless brain stimulator. Response was registered for each trial when both hands were raised. Body movements were detected by a time-of-flight camera, Kinect^TM^. (**b**) Examples of feedback on correct and incorrect responses. The game was adaptive to children’s performance; every 3 consecutive correct answers promoted the following trial to a more difficult level and vice versa.

Here, we compared the effects of combining tRNS during cognitive training and cognitive training alone on the learning of children with MLD. We targeted MLD, as it is under-researched compared to learning disabilities with similar prevalence and socioeconomic impact^12^. We used tRNS, as growing evidence suggests that it promotes steeper learning and training within the numerical domain in healthy adults compared to cognitive training alone^9, 13, 14^. Moreover, tRNS effects were more pronounced with increased arithmetic difficulty^15^, a condition akin to the daily struggles of children with MLD. We applied tRNS over children’s bilateral dlPFCs (F3 and F4 based on the International 10–20 system mapped on a wireless cap, Fig. 1a), shown to be implicated in mathematical learning in neuroimaging^16^ and tRNS studies on healthy adults^9, 15^. To examine the practical relevance of tRNS effects, we conducted our study in a school specialized for learning disabilities, and used training and transfer tasks that reflect real-world learning. Children were diagnosed by their school as having MLD based on persistent underachievement in mathematical performance given their age, cognitive abilities and educational experiences (see Supplementary Table S1, Fig. S1). The current cognitive training was on an adaptive number line game. Children moved their body from side-to-side to map the location of a given number on a virtual number line, and registered their responses by raising both of their hands (Fig. 1a). Our training featured number line mapping, a key predictor of mathematical achievement^17^, which correlates with arithmetic processing even in adults^18^. Its difficulty levels were automatically adjusted based on children’s performance. Children received feedback on their response in each trial (Fig. 1b). They were pseudo-randomly assigned to receive either “active” or sham tRNS throughout training based on their mathematical performance and WM, which were assessed at school before the start of the experiment (Table 1). We collected data on children’s performance during training, and on cognitive tasks [Mathematical Assessment for Learning and Teaching, (MALT) and working memory (WM)] before and after training. We also recorded their experiences of tRNS using a questionnaire at the end of the study. Based on previous studies on young healthy adults^9, 13, 15^, we hypothesized that compared to training alone, tRNS during cognitive training would (1) improve the learning of children with MLD and (2) increase transfer effects to a real-life task.

**Table 1:** Children with MLD were matched across groups (sham vs. tRNS) by their age, gender, mathematical abilities, and working memory capacity.

**Controlled variable**	**tRNS**	**sham**	**Differences between groups**
Age (months) Mean (SD)	114.8 (6.6)	112.8 (8.5)	t(10) = 0.46, *p* > 0.66
Gender	5 boys, 1 girl	5 boys, 1 girl	χ²(1) = 0, *p* = 1
Mathematical performance Mean (SD)	13.5 (8.2)	13.2 (5.5)	t(10) = 0.08 *p* > 0.94
Equivalent Mathematical Age (SD)	87.8 (15.2)	88.8 (10.6)	t(10) = −0.13, *p* > 0.9
Working memory Mean (SD)			
Verbal			
Forward	4.67 (0.52)	4.17 (0.98)	t(10) = 1.1, *p* > 0.3
Backward	2.67 (0.81)	2.5 (0.55)	t(10) = 0.41, *p* > 0.69
Visuospatial			
Forward	4.17 (0.98)	3.83 (1.33)	t(10) = 0.49, *p* > 0.63
Backward	3.0 (0.63)	3.0 (0.89)	t(10) = 0, *p* = 1
Guessed condition	6/6 real	6/6 real	χ²(1) = 0, *p* = 1

While MALT provided the equivalent mathematical age based on raw scores, it did not provide a standardised score below a certain level of performance. Therefore, we presented the raw scores and the mathematical age equivalence. Working memory capacity is represented by standard scores.

## Methods

### Participants

Twelve children with MLD aged 8.5–10.9 (mean ± one standard error of the mean: 113.8 ± 7.3 months) without other neurological or psychiatric illness participated in this study. Children were selected from the same school, which was the only school that agreed to participate in our research at this stage given the lack of any records on the use and safety of tRNS for paediatric population. Children were diagnosed as having specific MLD by Fairley House School (London), a specialised school for children with specific learning needs. We trained children in the same room to control for any variance attributed to different learning environments. They were pseudo-randomly assigned to sham and active tRNS groups (Table 1). We obtained informed consent from parents/guardians, and children gave their assent to participate in this study. This study received ethical approval from the National Research Ethics Service Committee South Central-Hampshire B (11/SC/0401). All methods were performed in accordance with the approved guidelines and regulations.

### Cognitive training

Children moved their bodies from side-to-side to map numbers on a virtual number line ﻿presented on an interactive whiteboard. They registered their responses by raising both hands in front of a time-of-flight camera (Kinect^TM^) that detected body movements (Fig. 1a). Our training integrates bodily movements, as spatial-numerical and embodied learning have been suggested to promote better training effects in children than training without embodiment^19–21^. The game was adaptive, and challenged children with a range of difficulty levels depending on their performance. It featured number mapping of a range of difficulty levels (defined by the range of the number line; 0–5, 0–10, 0–50, 0–100, 0–500, 0–1000, and 0–1500) and a range of precision levels (7%, 6%, 5% and 4%) within each difficulty level. Three consecutive correct answers promoted the next trial to a more difficult level/precision level, while three consecutive incorrect answers demoted the next trial to an easier level/precision level (Fig. 1b). This game yielded three main measures: accuracy (precision of number mapping), response times (RT) and the level of each trial attempted. Children were instructed to move their body from side-to-side to locate the position of the number prompted on the center of the screen above the virtual number line, and to raise both their hands to register their answer. They were asked to respond as quickly and as accurately as they could for each trial. As the game included 7 difficulty levels and 4 precision levels, there were 28 levels in total. Children trained on this game for 9 days, 20 minutes each at school for 2 days per week, over the course of 5 weeks.

### Transcranial random noise stimulation (tRNS)

Children in the active tRNS group received 0.75 mA of tRNS (0.1–500 Hz) to their bilateral dorsolateral prefrontal cortices (dlPFCs) via two saline-soaked, 25 cm^2^ circular sponges, attached under designated electrode positions (F3, F4) of a wireless tRNS cap that followed the International 10–20 system (Neuroelectrics Inc., Barcelona). The dlPFC was chosen, as the bilateral dlPFCs have been implicated in numerical processing in children^22^, and tRNS to the dlPFC during arithmetic training has shown promising results in healthy adults^9, 15^. TRNS was applied for 20 minutes per session as in previous tRNS protocols^9, 15^ and studies on paediatrics using another form of tES, transcranial direct current stimulation (tDCS)^23, 24^. We ran our study for 5 weeks in total, having considered the practical limitations of collecting data from school; term time, school timetable, and commitment from teachers to accommodate our study requirements (children catching up with lessons after taking part in our training). A light, battery-operated device attached to the back of the child-sized cap delivered electrical current for 20 minutes throughout each training session. As there were no prior studies using tRNS in children, we followed the recommendation of applying at least half of that administered to adults^25^; we applied 75% of 1mA^9, 13, 26^, shown to be well tolerated in adults without adverse effects. This dosage is estimated to equal that of 1 to 1.5 mA in adults, based on previous modeling of tDCS^25^. This decision was made after considering the parameters that would influence current distribution and density at the site of stimulation such as thinner scalp, less cerebrospinal fluid, and smaller head size of the paediatric population^27, 28^. A similar dosage using tDCS was well tolerated by children, and was not associated with adverse effects^29^. Despite stronger results with stimulation on consecutive days shown in a previous study on adults^30^, stimulation was applied during training twice a week, as a cautious approach to prevent and minimise any potential adverse effect of tRNS on paediatric population^11^, which are unknown at this stage. Children in the control group who did not received tRNS (no stimulation at all) wore the wireless cap to control for any placebo effects associated with wearing the tRNS cap. All children were blinded to their stimulation condition.

### Mathematical assessment

Children’s mathematical performance were assessed using MALT, a standardized diagnostic tool calibrated to the UK National Curriculum levels^31^. MALT is commonly used by schools to monitor the performance of their students. Children were given 45 minutes to solve as many mathematical problems as they could, using pencil and paper. MALT was administered before and immediately after the 9 days of training.

### Working memory

Given the link between mathematical achievement and working memory^32^ and findings of a previous study using tDCS^33^, we assessed children’s verbal and visuospatial working memory capacity using digit span and Corsi blocks respectively. We also examined performance on these indices to monitor for any changes associated with training effects, as well as any potential cognitive side effects linked with stimulation^34, 35^. In the digit span task, children listened to a series of digits read aloud by the experimenter and were asked to repeat the sequence. The first trial consisted of 2 digits, and the span increased by one digit with every 2 trials. In the Corsi blocks task, children observed the experimenter tapped a sequence on a series of blocks, and were asked to repeat the tapped sequence. Similar to the digit span task, the first sequence consisted of 2 blocks, and the tap sequence increased by one block with every 2 trials. Each task was terminated by 2 consecutive incorrect trials. The standard score of each task was the maximum number of items recalled in 2 consecutive spans.

### Statistical analysis

In the main text, we report the results based on linear mixed effects models, which accounts for within-subject correlations more optimally compared to Analysis of Variance (ANOVA) i. For the interested reader, we also report the results of ANOVA and Bayesian analysis in the Supplementary Information, which show converging results. Due to our limited sample size however, we recommend these inferential statistics to be interpreted carefully.

#### Video game

Number line ranges increased as a function of Levels (e.g., Level 1: 0–5, Level 2: 0–10, Level 3: 0–50 etc.). To allow for comparison across Levels, we standardised accuracy responses across all Levels [Absolute deviation from Target/Number range*100], in line with previous studies^17^. We log-transformed (natural log) these scores twice to achieve a normal distribution of the residuals in mixed effects model analysis. For the ANOVA analysis in the Supplementary Information, these scores were log-transformed only once.

#### Transfer effects

As a measure of the accuracy of training performance, we used the normalised absolute percentage error as specified by the equation in the previous paragraph. We calculated the rate of gain in these accuracy values over the 9 days for each subject according to the following equation: [(Day 9–Day 1)/Day 1] and correlated it with children’s rate of improvement in MALT measures using the following equation: [(Post-training score − Pre-training score)/Pre-training score]*100.

#### Accuracy, Overall Levels and Response Times (RT)

We used R (R Core Team, 2016) and *nlme* ^36^ to perform a linear mixed effect analysis with maximised log-likelihood on the accuracy, overall levels and mean RT. The limits of the 95% of the bootstrapped distribution (R = 10,000) of beta coefficients were obtained using the package *boot* ^37^ [bootstrapped percentile interval, BPI). We set Group, Day (as continuous variable) and the interaction Group x Day as fixed effects and random intercepts for participants.

#### The effect of training on mathematics assessment

We then used regression analysis to examine how Group (tRNS vs. sham) affected the transfer from training to mathematics performance. This analysis was performed using Amos 22 (Arbuckle, 2013) using the maximum likelihood estimator.

### Children’s experience of tRNS

Children were given a questionnaire at the end of training (9^th^ Day), in which they were asked the following questions:

1. Did you receive brain stimulation? (A. During all sessions; B. Sometimes; or C. Not at all).
2. Do you think wearing the cap helps you to do well in maths? (A. Yes; B. Maybe; or C. Not at all). Why?
3. How did you feel when you wore the hat? (Open-ended question).
4. Did you feel uncomfortable wearing the hat? (A. Yes; B. Sometimes; or C. Not at all).
5. If the hat can help you with maths, will you wear it? (A. Yes; B. Maybe or C. Not at all).

### Long-term effects

To assess for any long-term effects of tRNS, a one-off assessment of performance on the training and MALT was conducted without tRNS four months later (see Supplementary Results).

## Results

### Performance in cognitive training

#### Accuracy

**Figure 2:**
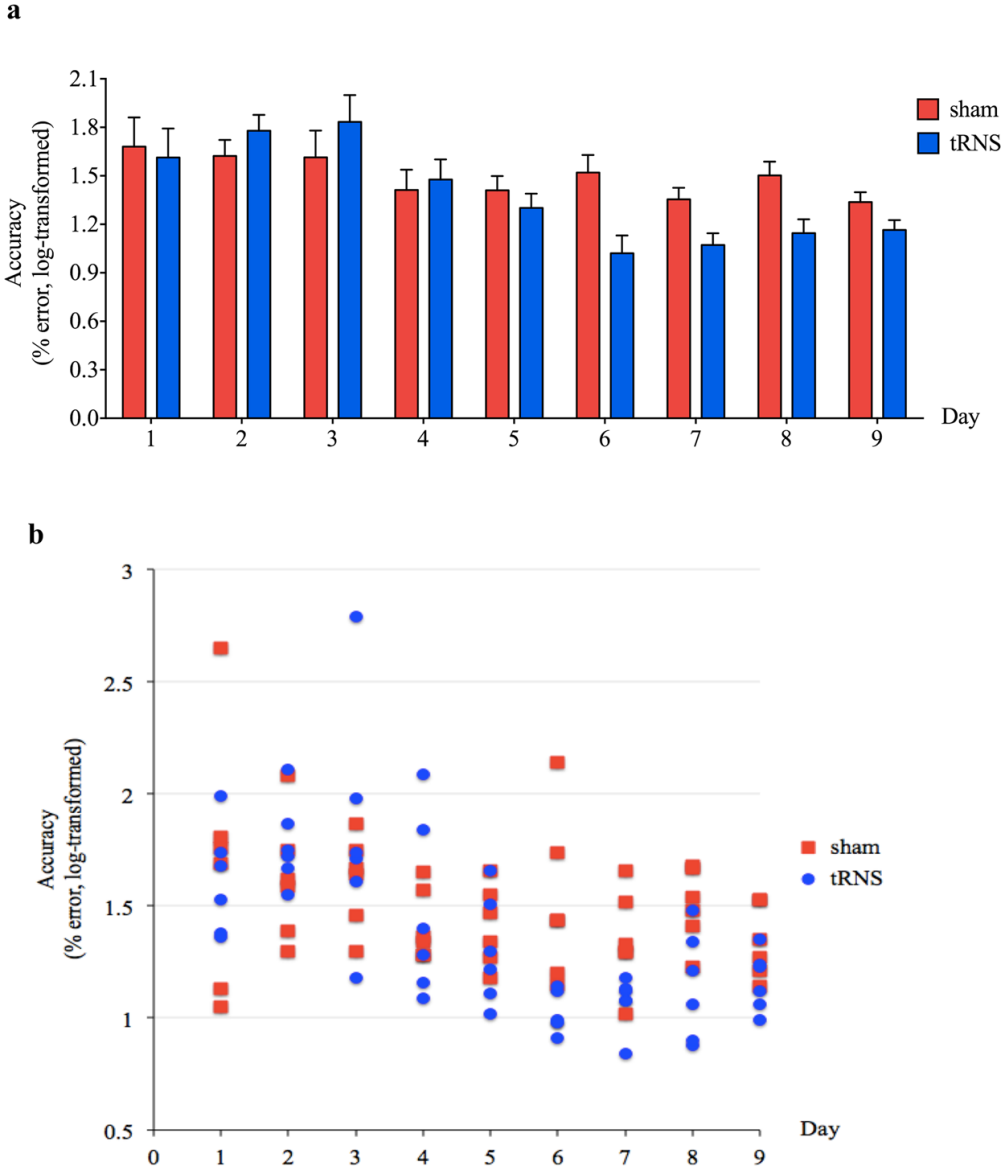
Improved accuracy with transcranial random noise stimulation (tRNS). Only children in the real tRNS group showed improved accuracy with training. Those in the sham group showed little improvement, characteristic of those with learning disabilities. (**a**) Bar graph indicates the mean accuracy of each group on each training day. Error bars indicate one standard error of the mean (SEM). (**b**) Scatter plot displays the mean accuracy of each individual on each training day, labelled according to group.

The main effect of Day was statistically significant (B = −0.02, SE = 0.008, 95%BPI = [−0.036, −0.005], *p* = 0.012) whereas the main effect of the Group was not (B = 0.13, SE = 0.09, 95%BPI = [−0.01, 0.27], *p* = 0.183). Crucially, the interaction between the Group and Day was statistically significant (B = −0.046, SE = 0.012, 95%BPI = [−0.07, −0.02], *p* < 0.001), thereby suggesting that the decrease of error as a function of day was larger for the real tRNS group compared to the sham group (Fig. 2). The residuals of the model were normally distributed as confirmed by visual inspection and a non-significant Shapiro test (*p* = 0.62).

#### Overall Levels

**Figure 3:**
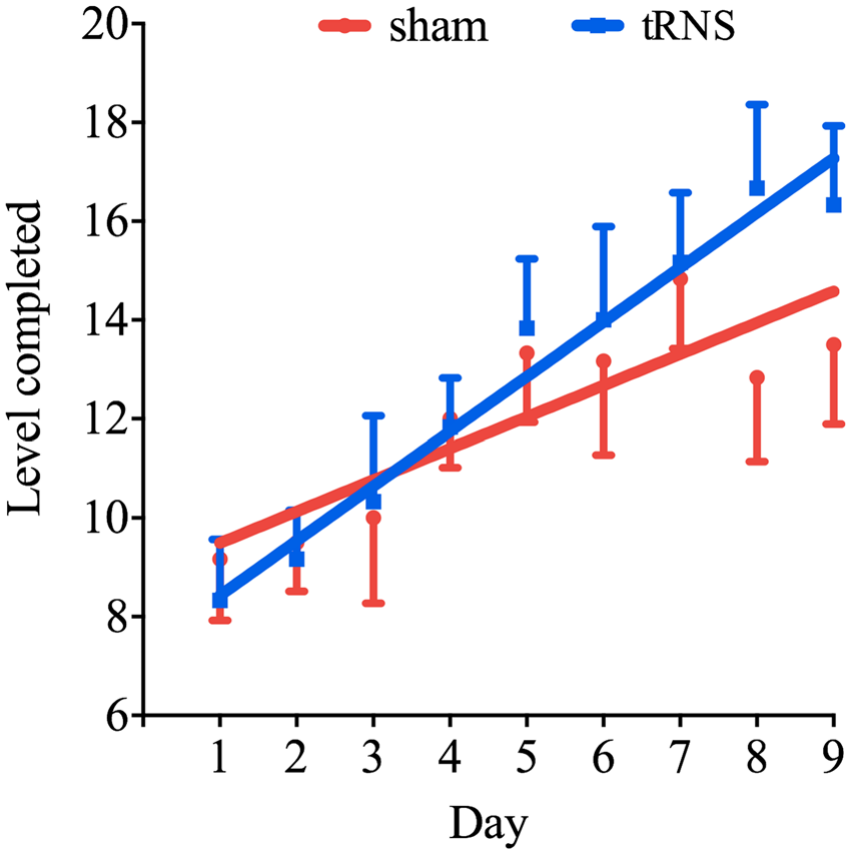
Steeper learning with tRNS. Children in the real tRNS group showed a steeper learning slope compared to those in the sham group. Data points indicate the averaged group level at the end of each training day. Error bars indicate one standard error of the mean (SEM).

The main effect of Day was statistically significant (B = 0.64, SE = 0.13, 95%BPI = [0.41, 0.87], *p* < 0.001) whereas the main effect of Group was not (B = −1.53, SE = 1.75, 95%BPI = [−3.39, 0.27], *p* = 0.402). Notably, the interaction between Group and Day was statistically significant (B = 0.47, SE = 0.18, 95%BPI = [0.13, 0.82], *p* = 0.01), suggesting that the increase in achieved level as a function of day was larger for the real tRNS group compared to the sham group (Fig. 3). The residuals of the model were normally distributed as confirmed by visual inspection and a non-significant Shapiro test (*p* = 0.62).

#### Response times

**Figure 4:**
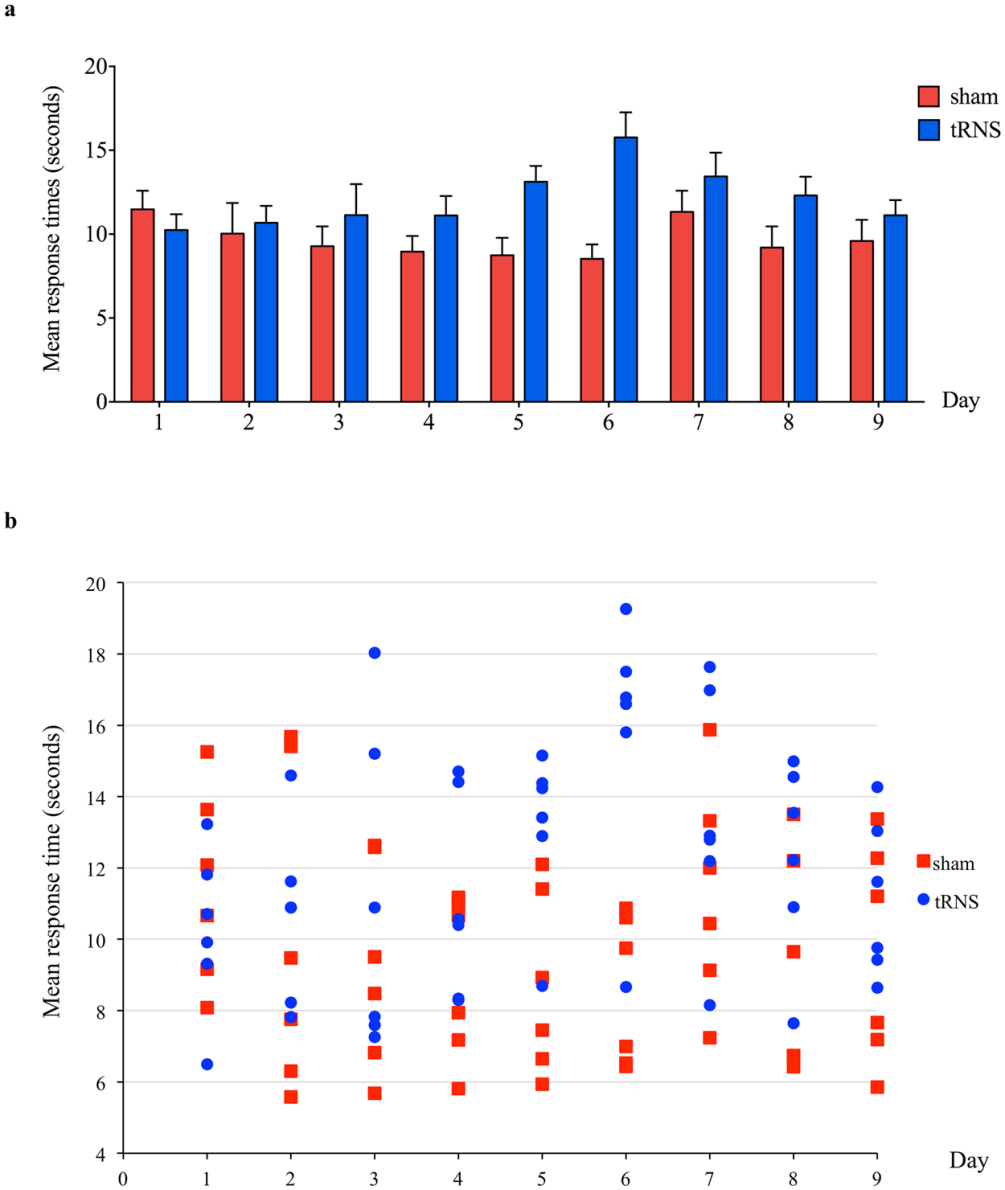
Sham group showed reduced response times with day compared with the real tRNS group, but was not associated with speed-accuracy trade-off (**a**) Bar graph displays the mean response time of each group on each training day. Error bars represent one standard error of the mean (SEM). (**b**) Scatter plot shows the mean response times of each individual on each training day, labelled by group.

The main effect of Day (B = −0.11, SE = 0.13, 95%BPI = [−0.31, 0.11], *p* = 0.405) and the main effect of Group did not reach significance (B = 0.42, SE = 1.5, 95%BPI = [−1.66, 2.65], *p* = 0.788). However, the interaction between Group and Day was statistically significant (B = 0.40, SE = 0.18, 95%BPI = [0.04, 0.78], *p* = 0.028). The response times of the sham group tended to decrease slightly﻿, whereas the real tRNS group increased as a function of day (Fig. 4). The residuals of the model were normally distributed as confirmed by visual inspection and a non-significant Shapiro test (*p* = 0.11).

#### Speed accuracy trade-off

For each participant, we calculated the regression coefficients respectively for the mean response times and accuracy as a function of day. Then, we added the individual regression coefficients for response times as fixed factor into the previously run mixed effects model on accuracy and the individual regression coefficients for accuracy as fixed factor into the mixed effects model on response times. In both cases, the interactions between day and group remained significant.

Please see Supplementary Information for ANOVA and Bayesian analyses that show similar results.

### Working memory

We performed a linear mixed effects analysis on working memory scores with Time (pre- and post-training), Task (Verbal WM, Visuospatial WM), Order (Forward, Backward) and Group (tRNS, sham) as fixed effects and random intercepts for participants. There were no significant interactions with Group (all *p* > 0.35).

### The effect of training on mathematics assessment

We found a positive correlation between percentage change in accuracy during training and change in age-equivalence in mathematics based on the MALT [non-parametric (Spearman) correlation: *r*_*s*_ = 0.75, *p* = 0.005, 95% confidence intervals (CI) with bias-corrected and accelerated percentile bootstrap method with 10,000 resampling to exclude potential effects of outliers [0.12, 0.97]] (Fig. 4a). In contrast, the correlation between RT and MALT was not significant [*r*_*s*_ = 0.23, *p* = 0.47, 95% CI [−0.53, 0.83]]. The same correlation between accuracy and MALT was significant when we analysed raw scores instead of age-equivalence in mathematics, or parametric (Pearson) correlation analysis (all *r* > 0.64, all *p* < 0.03). This consistent relationship indicated that greater improvement in accuracy, which was improved by tRNS was correlated with improved maths performance.

To understand how coupling tRNS with cognitive training affected MALT scores (i.e., transfer effect), we constructed a path model (Fig. 4b). We predicted that improvement in accuracy, which was observed for Days 6 to 9 for tRNS group only (see ANOVA results in Supplementary Results), would have translated to higher MALT scores. Prior to this set of analyses, we *z*-standardized all scores (to allow for inference of effect sizes) and created the interaction term from these standardized scores.

As predicted, in Model 1 (Fig. 4b), Group was not associated with gain in accuracy scores across Days 1 to 5 (*β* = 0.11, *p* = 0.71) or change in MALT scores (*β* = 0.31, *p* = 0.28). Furthermore, gain in accuracy across Days 1–5 was not associated with gain in MALT (*β* = −0.05, *p* = 0.87), and Group did not moderate the relationship between gain in accuracy across Days 1 to 5 and gain in MALT scores (*β* = −0.27, *p* = 0.32).

**Figure 5:**
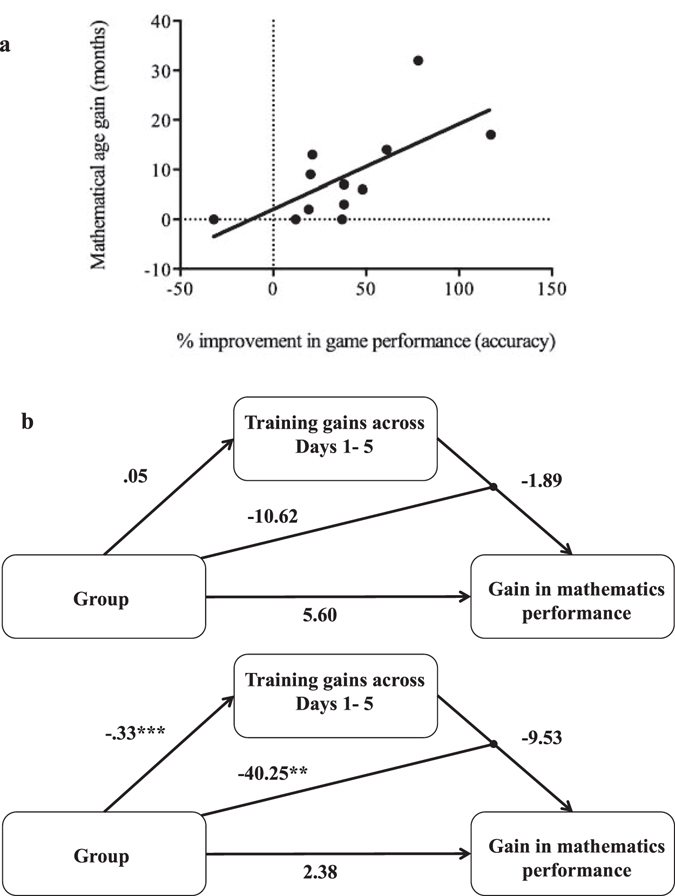
Transfer of training gains to real-life achievement (**a**) Improvement in training transferred to performance on a standardised diagnostic mathematics test (MALT), a measure of achievement at school. Note that while we presented the actual values, the correlation was calculated using rank data (Spearman correlation) to account for the sample size and to avoid spurious results due to outliers. These points are not labelled by groups, as we found no effect of group. (**b**) Model 1 shows the non-significant effect of tRNS on training across Days 1 to 5 and gain in maths performance as indicated by the MALT (mathematical age-equivalence). Model 2 presents the significant relationship between gains in accuracy across Days 6 to 9 and gain in maths performance as indicated by the MALT (mathematical age-equivalence), which was moderated by group (tRNS vs. sham). Models 1 and 2 demonstrate the influence of tRNS on transfer, which depend on its effect on cognitive training. For the sake of transparency, we presented the unstandardised regression coefficients in the figure, and reported the corresponding beta weights in the text. ** = *p* < 0.02, *** = *p* < 0.001.

In contrast, in Model 2, Group was negatively associated with gains in accuracy across Days 6 to 9 (*β* = −0.77, *p* = 0.001), indicating that children who received tRNS showed larger gains in accuracy between Days 6 to 9 compared to those in the sham group. Group was not significantly directly associated with MALT scores (*β* = 0.13, *p* = 0.77). Similarly, the simple main effect of gains in accuracy across Days 6 to 9 was not significantly associated with MALT scores (*β* = −0.22, *p* = 0.62). Importantly, we found that Group moderated the relationship between gains in accuracy across Days 6 to 9 and gain in MALT (*β* = −0.99, *p* = 0.01, Fig. 5). We used an online interaction tool^38^ to decompose the interaction term. Simple slopes analysis indicated that the relationship between gains in accuracy across Days 6 to 9 was associated with improved MALT scores (i.e., transfer effect) only for the tRNS group (tRNS: *β* = −1.61, *p* = 0.02; sham: *β* = 0.28, *p = *0.6). The results were not altered when we included working memory scores as a covariate.

### 3.4 Children’s experience of tRNS

Children did not discriminate between active and sham tRNS; all children thought that they received stimulation [χ²(1) = 0, *p* = 1] and none thought that wearing the cap did not help. These responses suggested effective blinding, despite our single-blind design. This is in line with previous studies on adults, indicating the high cutaneous threshold of tRNS^15, 26^ and possibly, the lower current used in our study (0.75 mA). When asked if they thought wearing the cap helps, 4 out of 6 children in the tRNS group responded ‘Yes’, 5 out of 6 children in the sham group responded ‘Yes’, and the rest answered ‘Maybe’ (χ²(1) = 0.44, *p* = 0.51). From the tRNS group, one child felt uncomfortable wearing the hat and three children mentioned that sometimes they felt uncomfortable. From the sham group, two children mentioned that an uncomfortable feeling occurred sometimes (χ²(1) = 1.33, *p* = 0.25). This discomfort was attributed to itchiness caused by the strap around the chin. When asked if they would wear the cap if it could help them with maths, all children in the sham group answered ‘Yes’, while those from the tRNS gave varied responses (Yes: 3/6, Maybe: 2/6, No: 1/6). Notably, the child who answered “No” from the tRNS group explained that he did not want to look weird). Multinomial logistic regression indicated that there is no statistical difference between groups (Wald chi-square = 5.18, *p* = 0.8). Overall, children’s responses did not indicate any physical side effects that they attributed to tRNS. As the evidence so far on the relative safety of tES for children has been mainly based on the lack of adverse effects using tDCS^27, 39–41^, these responses provide preliminary data on the tolerability of tRNS for this population.

## Discussion

In the current study, we investigated whether compared to training alone, tRNS during cognitive training would (1) improve the learning of children with MLD and (2) increase transfer effects to a real-life task.

Our preliminary data suggests that children with MLD who received tRNS showed more accurate performance across training days compared to those who received sham tRNS. This trend resembles that of a previous study on healthy adults that showed active tRNS over bilateral dlPFCs improved arithmetic performance compared to sham tRNS^9^. More generally, we found that tRNS was associated with a steeper learning rate compared to sham, corroborating previous findings on healthy adults within the mathematical^9, 13, 15^ and non-mathematical domain^42^.

Aside from improved training outcomes, there is an indication of a positive transfer from training to children’s achievement on MALT, a standardized diagnostic test that is calibrated to the national curriculum. Similar to findings of cognitive training involving healthy children in another cognitive domain^43^, the greater the training outcome, the more children improved in their mathematics performance. Notably, this transfer appear to be modulated by tRNS, consistent with accumulating evidence suggesting that tRNS during training increases the transfer of numerical training gains to related materials amongst healthy adults^9, 13, 15^ (Fig. 4b). Similar to tRNS studies on healthy adults^9, 13, 15^, we did not find transfer effects on working memory, suggesting the absence of transfer beyond trained domain^44^. Given the link between training gains and standardised diagnostic tool, these findings may offer implications for special education.

Of potential value for future replications, our study offers lessons on the practical difficulties and sample size constrains common to early-stage innovative clinical and translational research^45^. At the time of our study, the stimulation software was not developed with a double-blinding function. With limited resources, it was not feasible to conduct a double-blind study, as the sole experimenter was required for study design, participant recruitment, selection, and testing with the support of the school’s occupational therapist. However, the current data is unlikely to have been biased by the experimenter for three reasons. First, training was computerized and therefore limited the involvement of the experimenter. Second, observed behavioural changes did not occur at the beginning of training but rather, towards the end. Third, the observed improvement was not general; it was specific to maths, and was not observed in working memory performance. Given the novelty of tRNS for paediatric use and hence, the lack of safety records and scientific precedent of its application (especially in a cognitive training context, where tRNS is applied for longer, i.e., more than a single session), the lack of collaborations with other schools prevented the recruitment of a larger sample size at this stage. This contributed to the practical difficulties of power-based sample size planning, common to novel experimental approaches^46^. Nonetheless, it has been suggested that neglecting such practical difficulties in early-stage research may pose barrier to innovation and translation^45^. As part of our effort to reduce error variance, we controlled for several parameters across groups in our study and conducted recruitment and training in the same school (Table 1). Given the exploratory nature of the current study, these preliminary evidence encourage further replications using larger samples with greater power^47^.

Overall, the current study contributes insights into the novel concept of combining tRNS and cognitive training for mitigating developmental disorders. This approach addresses the ethical dilemma of depriving a considerable population of children of potentially improved cognitive abilities, which could have important individual and socioeconomic implications^5^. These experimental data is a first step towards addressing current debates that lacked empirical evidence, while raising open questions that are fundamental to understanding the workings and potential of brain stimulation use on children^11, 48^. Addressing these questions would require systematic replications involving randomised double-blinded controlled studies on larger samples, different educational/cultural systems, physiological data on the underlying neural and cognitive mechanisms^49^, and children with other atypical developmental conditions^5, 50^. Cognisant of the growth in public interest and do-it-yourself (DIY) application of brain stimulation for cognitive enhancement^51^, we emphasize that the current early-stage findings serve as impetus for further scientific investigations, but not support or justification for tRNS application outside the experimental context. While we did not find any evidence that would raise safety concerns, children in our sample were carefully selected and strictly assessed for their eligibility to receive brain stimulation. Randomised-controlled studies with larger sample sizes that are more likely to be achieved by multi-site collaborations, would be more sensitive to detect any side effects attributed to other forms of non-invasive brain stimulation^34^. The potential and value of tRNS as a tool for understanding and intervening with atypical neurodevelopmental conditions such as learning disabilities warrants further research.

## Electronic supplementary material

Supplementary Information
