# Supplementary material for: Transcranial random noise stimulation and cognitive training to improve learning and cognition of the atypically developing brain: A pilot study
Source: Sci Rep. 2017 Jul 5;7:4633. doi: 10.1038/s41598-017-04649-x (PMC5498607; doi:10.1038/s41598-017-04649-x)
Supplement: Supplementary file 1 — Supplementary Information [file 41598_2017_4649_MOESM1_ESM.pdf]

Transcranial random noise stimulation and cognitive training to improve learning and cognition of the atypically developing brain: A pilot study

Chung Yen Looi[1], Jenny Lim[2], Francesco Sella[1], Simon Lolliot[1], Mihaela Duta[1], Alexander Avramenko[3], and Roi Cohen Kadosh[1*]

[1] Department of Experimental Psychology, University of Oxford, Oxford, OX2 3UD, UK

[2] Fairley House School, London SW1P 4AU, UK

[3] Cavendish Laboratory, University of Cambridge, Cambridge, CB3 0HE, UK

Supplementary Information

Supplementary Table S1.

Before the current study, children were diagnosed as having MLD by their school based on underachievement in mathematical performance given their age, cognitive abilities and educational experiences (see below). Verbal Comprehension and working memory (WM) performance was based on the subtests of Wechsler Intelligence Scale for children, 4th UK Edition (WISC-IV, UK). Mathematical ability was based on Numerical Operations subtest of the Wechsler Individual Achievement Test, 2nd UK Edition (WIAT-II, UK). As some information was not available and to assess children's abilities at the time, children's mathematical performance and WM

were examined at school prior to the start of the experiment, which determined group allocation (see Table 1).

Real tRNS (allocated based on criteria reported in Table 1)			
Subject	Verbal comprehension	Working memory	Mathematical ability
1	95	74	74
2	104	86	98
3	91	80	73
4	95	99	74
5	83	68	69
6	121	77	98
Sham tRNS			
7	100	91	66
8	112	88	Not available
9	96	83	77
10	128	91	81
11	93	74	<1 percentile
12	80	Not available	73

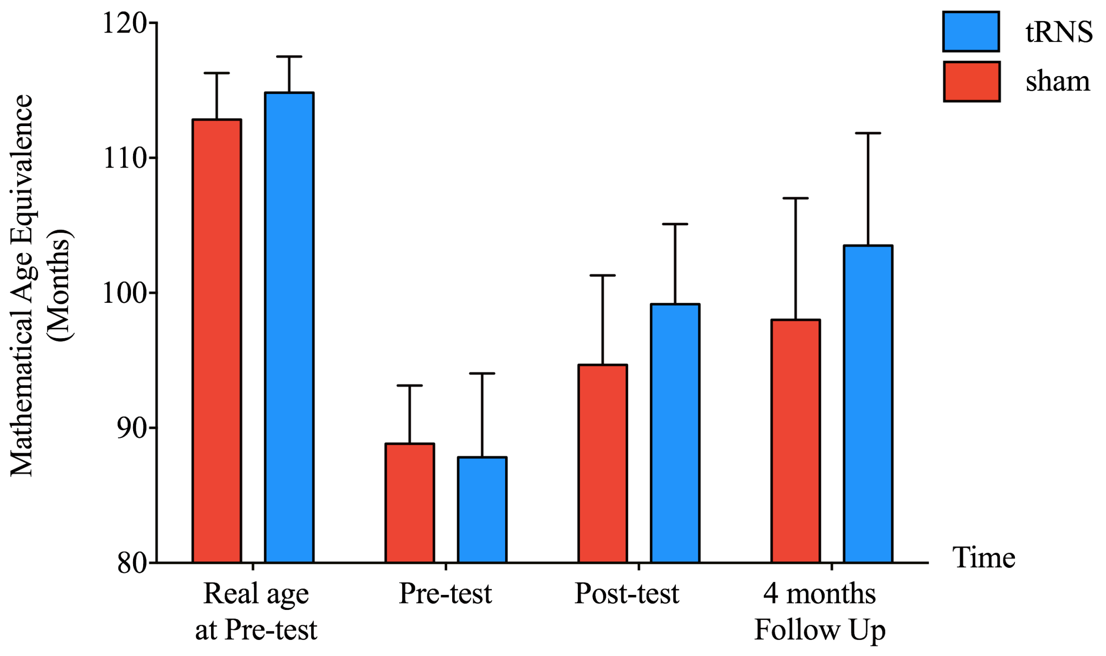

Supplementary Figure S1. Mathematical age equivalence of children based on the MALT across at pre-test, post-test and 4 months follow up.

Supplementary Results

Video game RTs, accuracy, levels completed, and working memory data were also analysed using Analysis of Variance (ANOVA) using IBM SPSS Statistics (significance= $p<.05$).

Performance on cognitive training

Accuracy A 2-way ANOVA was conducted with Time (Day 1-9) as within-subjects factor, and Group (tRNS, sham) as between-subjects factor. The interaction between Time and Group was significant [F(8,80)=3.69, MSE=.046, $p_{[GG]}$=.001013[<.001-=.008], η^2_p=.27]. This interaction stemmed from significant differences between tRNS and sham group from Day 6 onwards, indicating further improvements in the performance of tRNS group compared to sham after the first 5 training days (Day 6: *p*=.009; Day 7: *p*=.02; Day 8: *p*=.014; Day 9: *p*=.072; Fig. 2). When we decomposed this interaction by group, the sham group did not show a main effect of Time, suggesting a lack of significant improvement across training days [F(8,40)=2.01, MSE=.05, *p*=.07[<.001-=.482], η^2_p=.29, Observed power=.74]. In contrast, there was a significant effect of Time for the tRNS group, reflecting a steeper increase in accuracy with training [F(8,40)=12.33, MSE=.05, *p*<.001[<.001-<.001], η^2_p=.71, linear trend analysis explained 89% of the variance: F(1,5)=42.06, MSE=.076, *p*=.001[<.001-=.006], Observed power=1.0].

Response Times We ran the same two-way ANOVA for accuracy on the RT data. We found a significant interaction between Time and Group [F(8,80)=3.71, *p*=.001[<.001-=.015], η^2_p=.27, Observed power=.98]. This was due to significantly longer RTs on Day 5 [tRNS (13.12) vs. sham (8.74), *t*(10)=3.12, *p*=.011] and Day 6 [tRNS (15.76) vs. sham (8.53) , *t*(10)=4.19, *p*=.002]. Given the significant results on

accuracy, we examined whether the improvement in accuracy described in the previous section was due to a speed-accuracy trade-off [7]. We ran ANOVA with accuracy averaged over Days 6 to 9 (which have shown significant differences in the analysis above) as the dependent variable, Group as the independent variable, and RT (averaged over Days 6 to 9, as for accuracy analyses) as a covariate. There was still a main effect of Group [$F(1,9)=6.41$, MSE=.022, p=.032[<.001-=.88], η^2_p=.42; Observed power=.62], and importantly, RT was not a significant covariate (p>.42). These results indicated that the improvement in accuracy in tRNS group compared to sham is less likely to be attributed to RT.

Overall levels The differences between Groups in accuracy might stem from the possibility that those in the sham group reached higher and more difficult levels in the game and therefore, showed lower accuracy in mapping the numbers due to increased difficulty. To examine this, we assessed participants' performance based on the levels completed at the end of each training day. Linear trend analysis indicated that learning was steeper in the tRNS group than the sham group [$F(1,10)=5.9$, MSE=6.73, p=.036[<.001-=.938], η^2_p=.37, Observed power=.59] (Fig. 3).

To compliment the findings on the interaction between group and day on accuracy and overall levels achieved, we performed additional Bayesian analyses using the software, JASP[8] with default priors. First, we ran a mixed Bayesian ANOVA with Day as within-subjects factor and Group as between-subjects factor. We found that the interaction model was preferred to the main effects model by a Bayes factor (BF) of approximately 33. The analysis provided strong evidence in favour of the hypothesis that Day and Group interacted on accuracy. Subsequent Bayesian

independent *t*-tests revealed moderate evidence in favour of a better performance of the tRNS group compared to the sham group for Days 6 (BF=5.54), 7 (BF=3.32) and 8 (BF=4.07). Second, we ran a Bayesian independent *t*-test to compare the two groups on the mean of unstandardized beta coefficients obtained from the linear regression of completed levels as a function of day (i.e., linear trend). As previous results indicated steeper learning when tRNS was applied during cognitive training compared to sham stimulation within the mathematical[9-11] and non-mathematical domain[12], we considered a directional hypothesis with the tRNS group displaying a steeper learning compared to the sham group. We found moderate evidence (BF=4.31) in favour of the difference between the two groups. For transparency, the full output of the Bayesian analysis is provided below.

Output

Bayesian Repeated Measures ANOVA

Model Comparison - dependent

Models	P(M)	P(M|data)	BF $_M$	BF $_{10}$	% error
Null model (incl. subject)	0.200	4.382e -8	1.753e -7	1.000	
Day	0.200	0.047	0.197	1.072e +6	0.251
Group	0.200	2.268e -8	9.072e -8	0.518	1.813
Day + Group	0.200	0.028	0.117	648343.879	2.601
Day + Group + Day Group	0.200	0.925	49.057	2.110e +7	1.219

Note. All models include subject.

Bayesian Repeated Measures ANOVA

Model Comparison - dependent

Models	P(M)	P(M|data)	BF $_M$	BF $_{10}$	% error
Null model (incl. Day, Group, subject)	0.500	0.029	0.030	1.000	
Day Group	0.500	0.971	33.376	33.376	1.641

Note. All models include Day, Group, subject.

Analysis of Effects - dependent

Effects	P(incl)	P(incl|data)	BF Inclusion
Day Group	0.500	0.971	33.38

Bayesian T-Test

Bayesian Independent Samples T-Test

	BF_{10}	error %
S1_NAcc	0.478	2.947e -7
S2_NAcc	0.688	5.486e -5
S3_NAcc	0.616	3.410e -6
S4_NAcc	0.488	4.214e -7
S5_NAcc	0.596	1.325e -5
S6_NAcc	5.541	1.288e -7
S7_NAcc	3.316	9.298e -8
S8_NAcc	4.074	4.962e -8
S9_NAcc	1.453	1.129e -5

Inferential Plots

S1_NAcc

Prior and Posterior

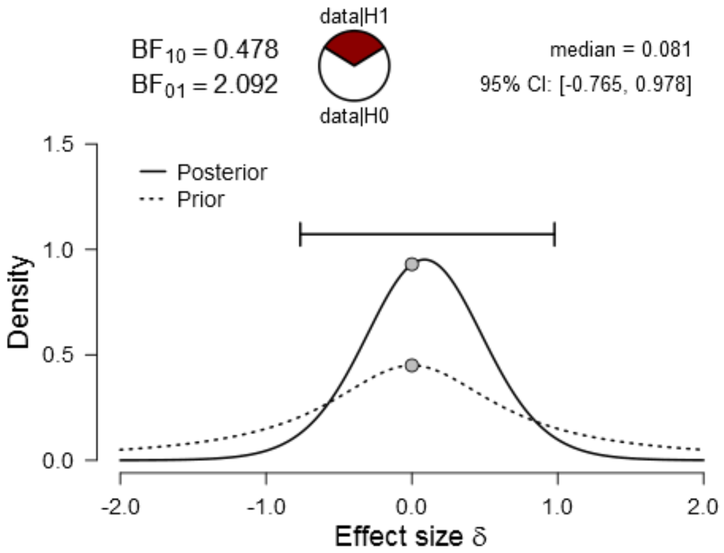

Bayes Factor Robustness Check

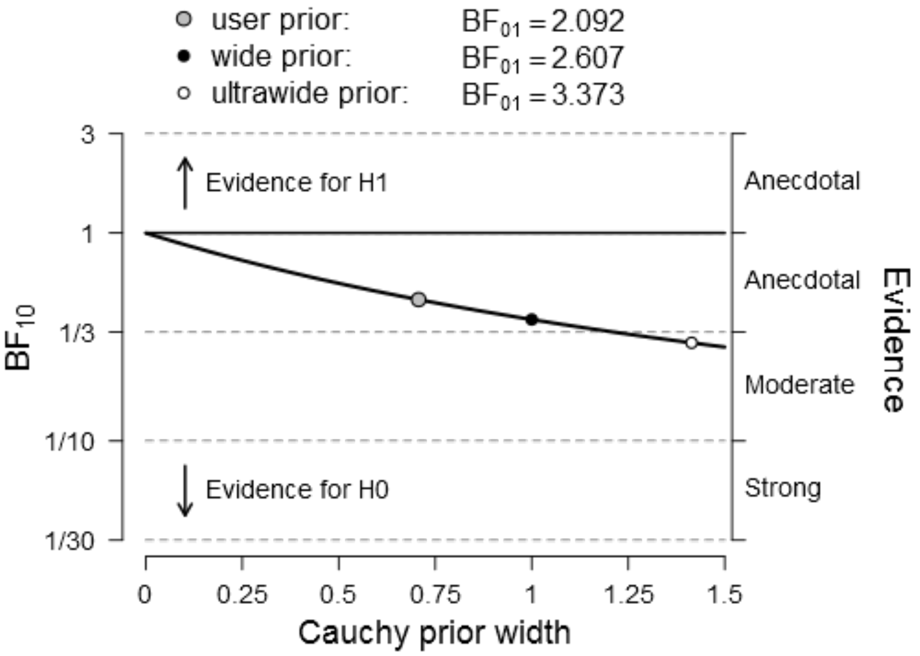

S2_NAcc

Prior and Posterior

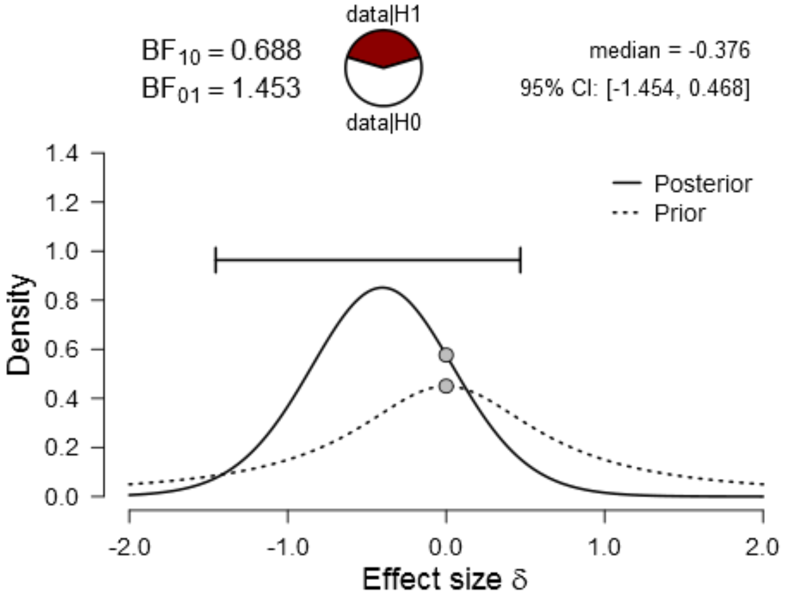

Bayes Factor Robustness Check

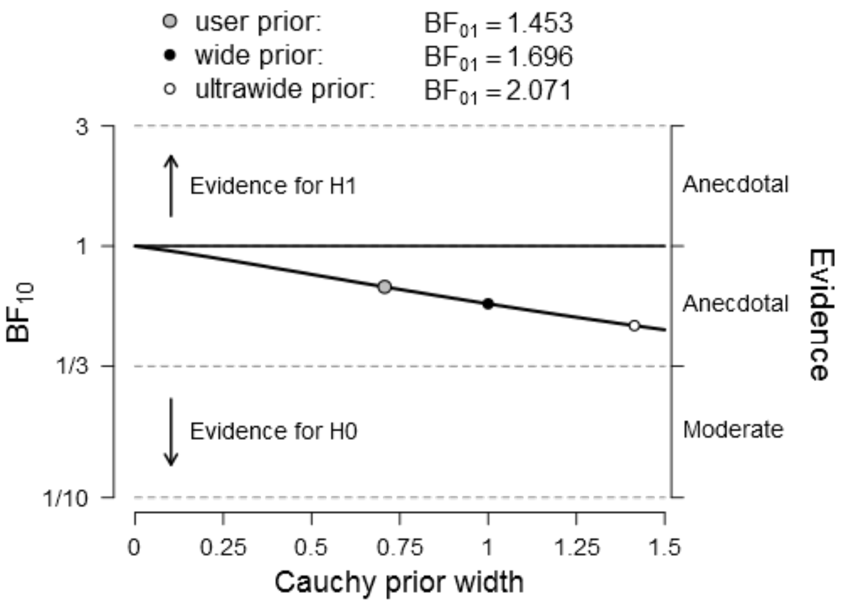

S3_NAcc

Prior and Posterior

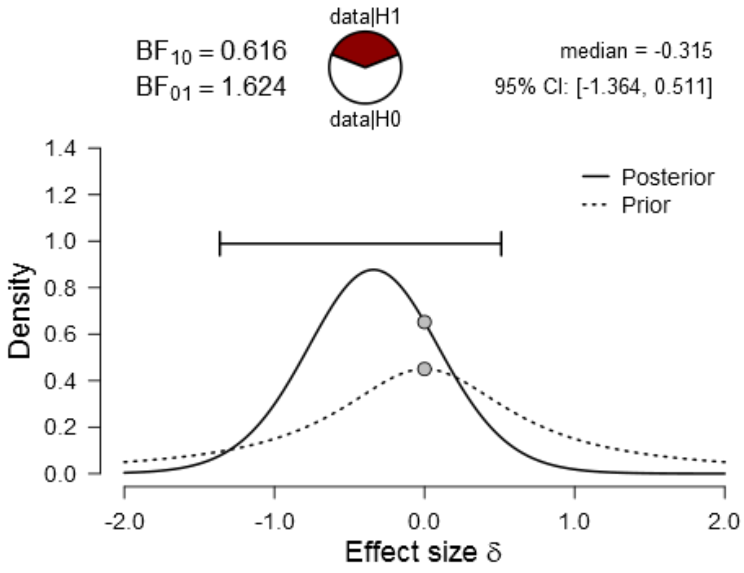

Bayes Factor Robustness Check

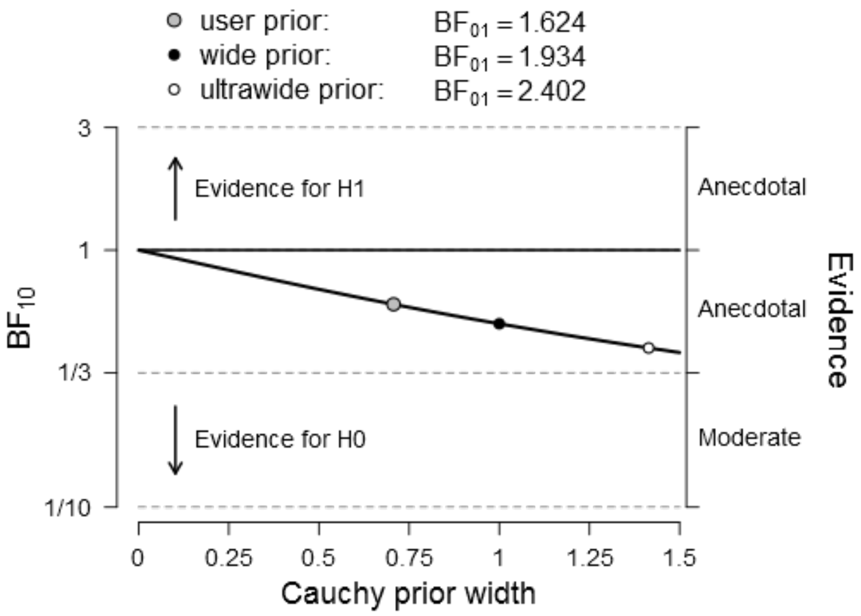

S4_NAcc

Prior and Posterior

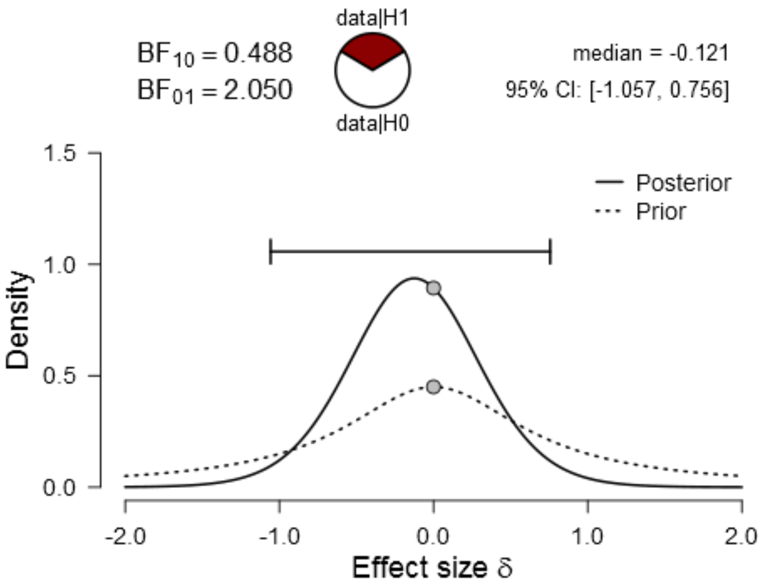

Bayes Factor Robustness Check

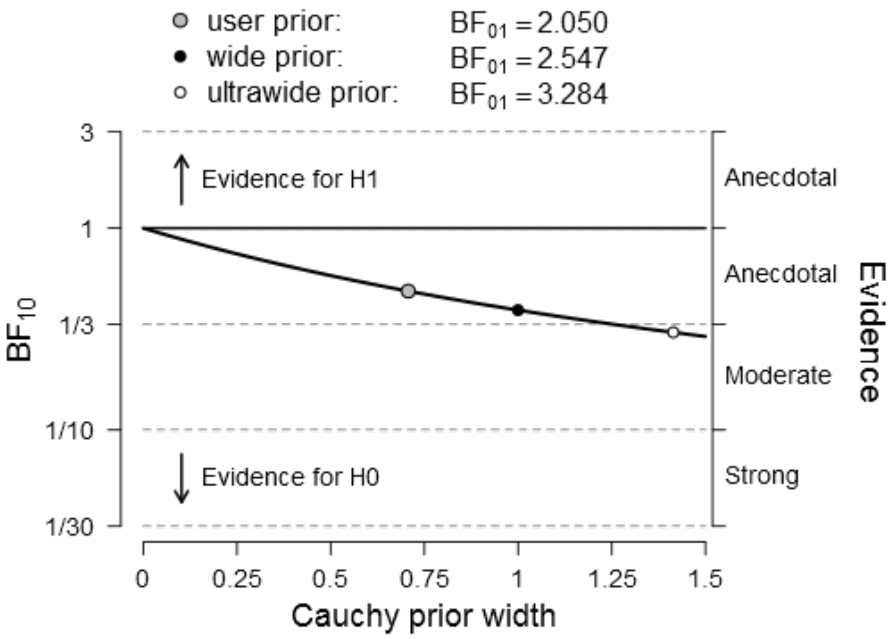

S5_NAcc

Prior and Posterior

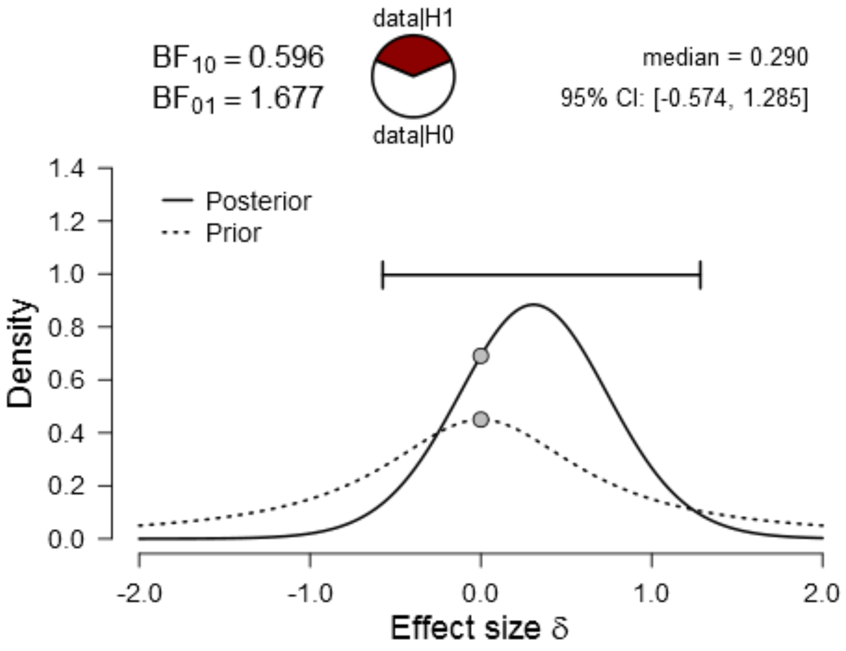

Bayes Factor Robustness Check

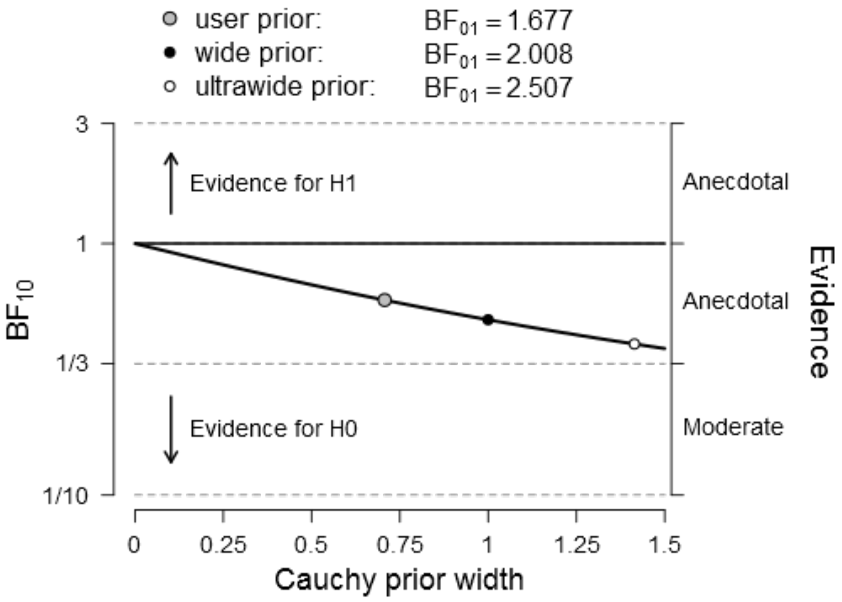

S6_NAcc

Prior and Posterior

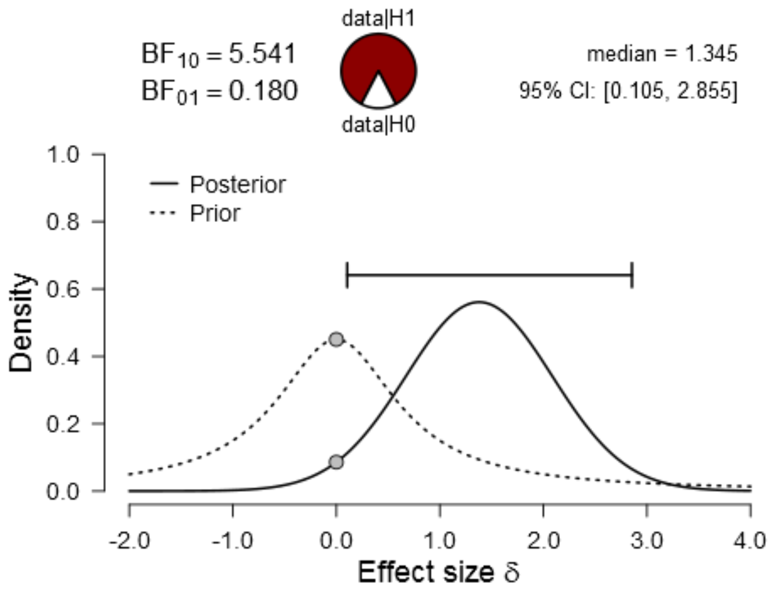

Bayes Factor Robustness Check

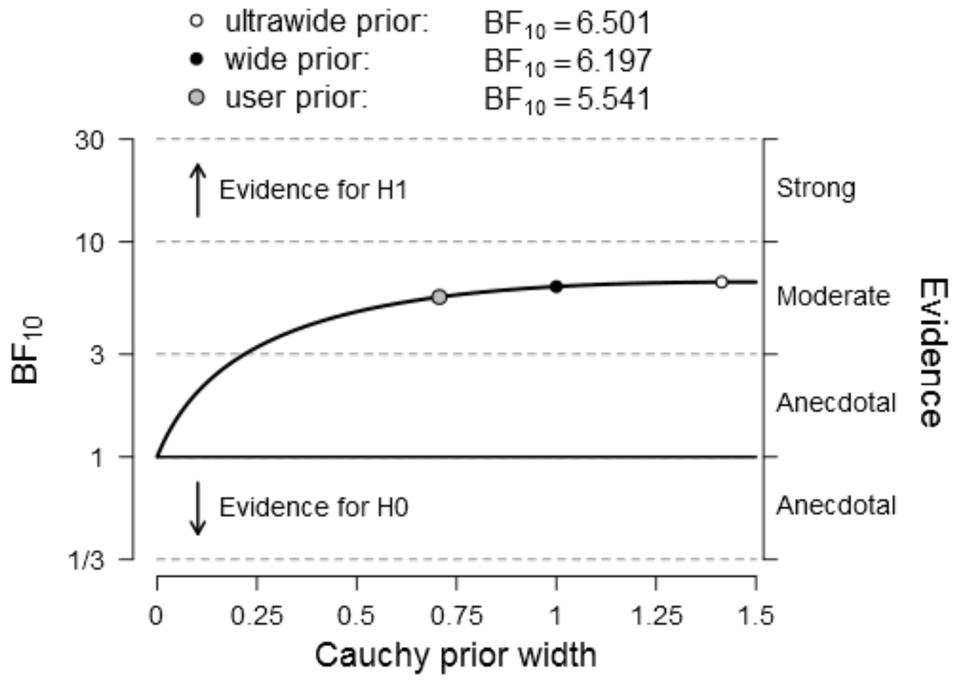

S7_NAcc

Prior and Posterior

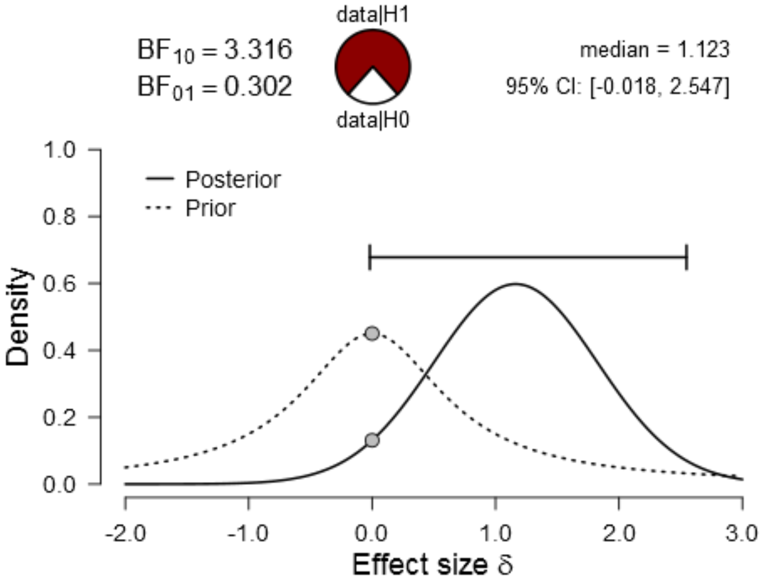

Bayes Factor Robustness Check

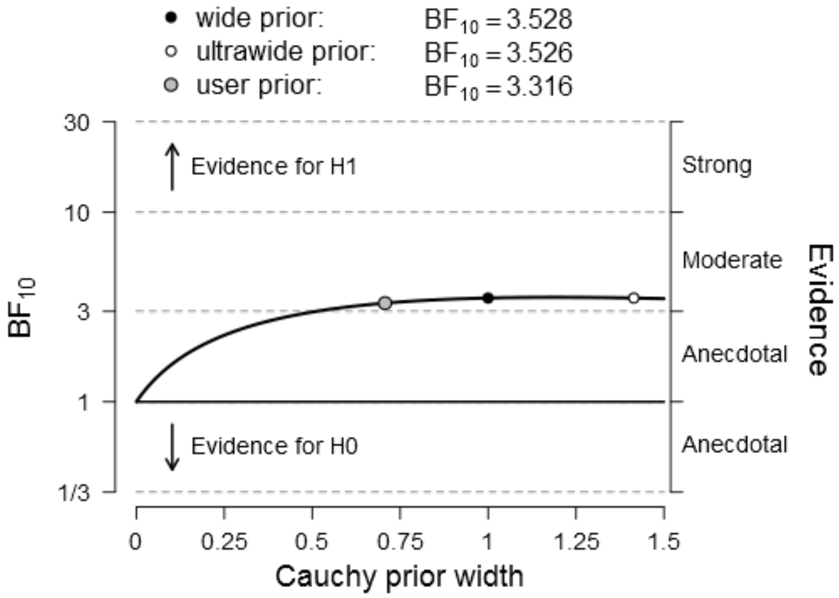

S8_NAcc

Prior and Posterior

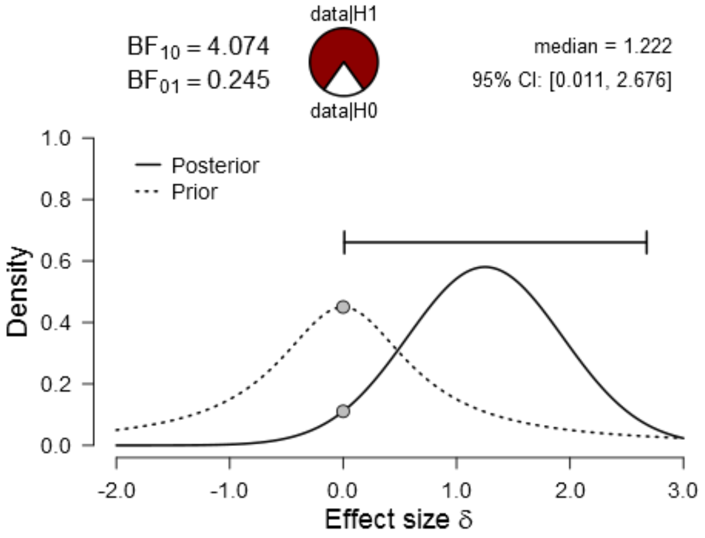

Bayes Factor Robustness Check

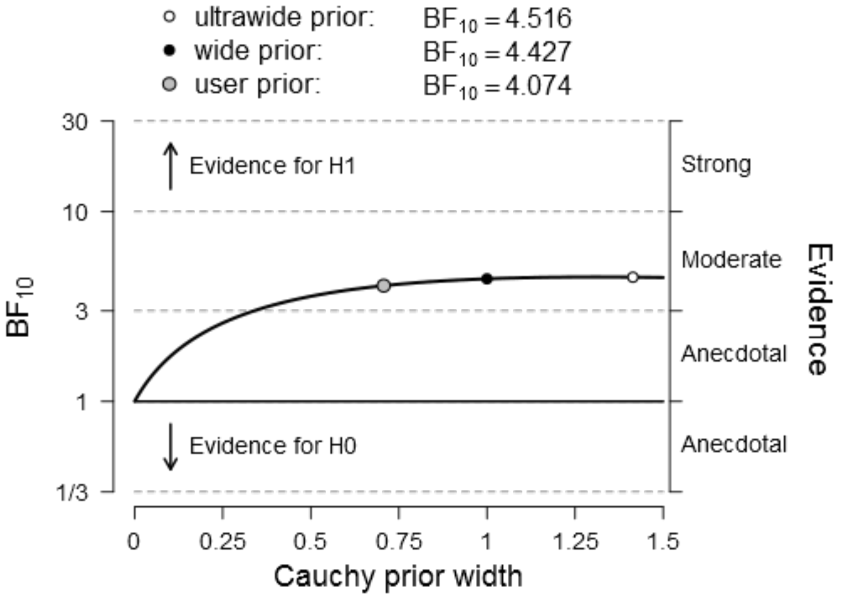

S9_NAcc

Prior and Posterior

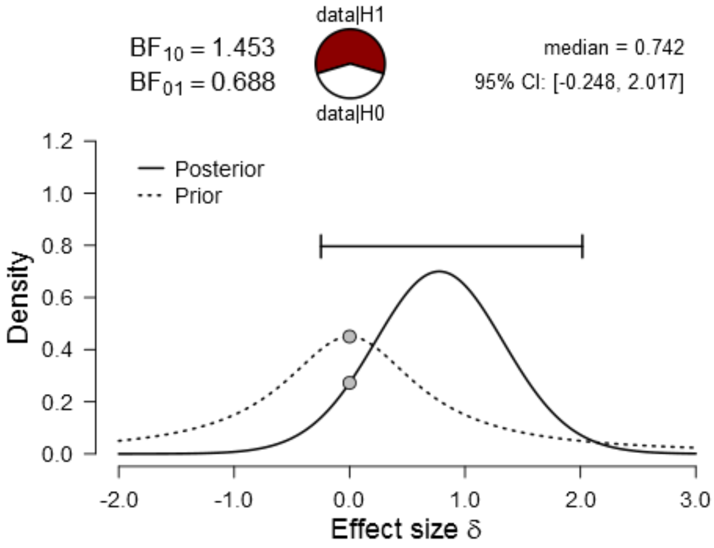

Bayes Factor Robustness Check

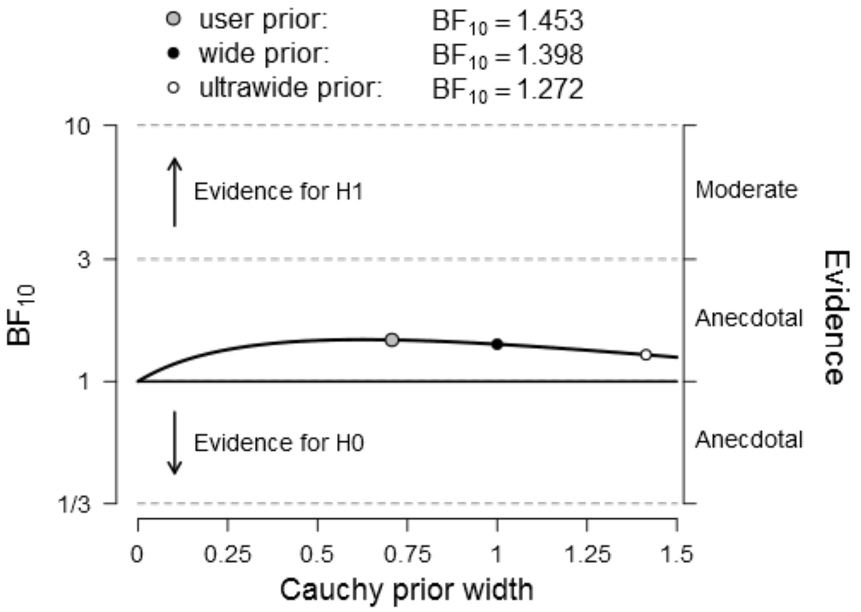

Bayesian T-Test

Bayesian Independent Samples T-Test

	BF_{-0}	error %
Betas	4.305	~ 1.042e -6

Note. For all tests, the alternative hypothesis specifies that group *Sham* is less than group *tRNS*.

Inferential Plots

Betas

Prior and Posterior

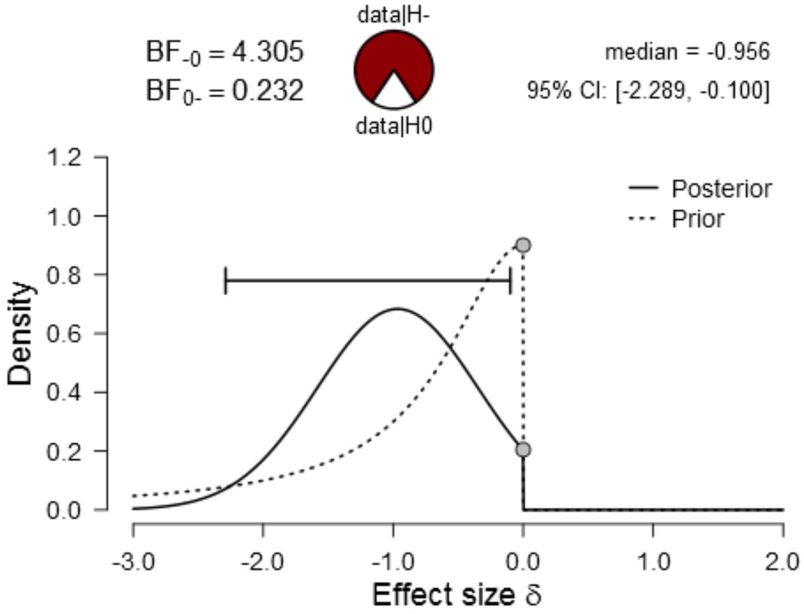

Bayes Factor Robustness Check

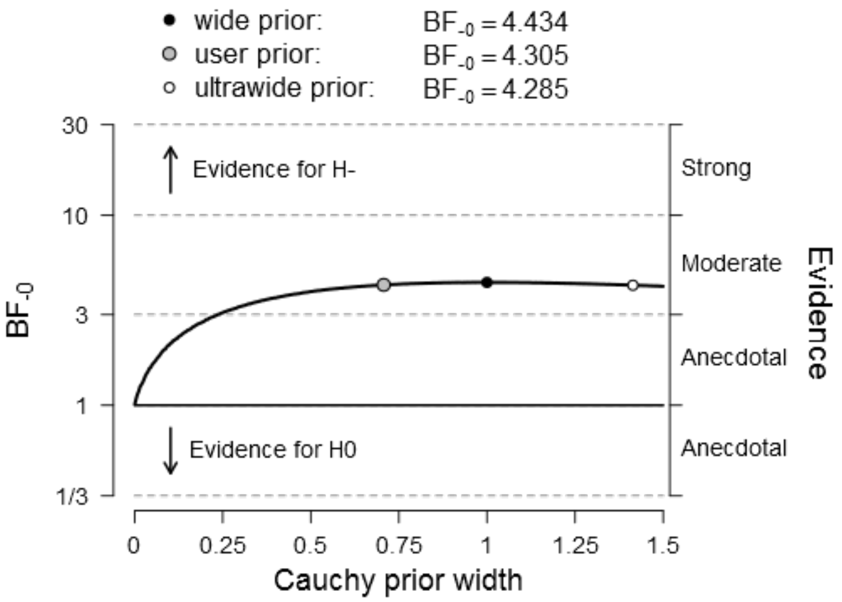

Long-term effects of training

At a 4-month follow-up (without further tRNS or cognitive training), we found a significant effect of Time [F(2,12)=6.24, MSE=43.3, p=.014, η^2_p=.51]. Linear trend analysis explained 53% of the variance [F(1,8)=9.09, MSE=46.8, p=.017; quadratic trend: F(1,8)=2.28, MSE=46.8, p=.17]. Improvement in MALT was sustained, and the expected maturation of 4 months from formal schooling was observed. Note that 2 children did not take part in the follow-up as they had changed school.

References

1 Chambers, C. D., Payne, J. M., Stokes, M. G. & Mattingley, J. B. Fast and slow parietal pathways mediate spatial attention. *Nat Neurosci* 7, 217-218 (2004).

2 Sack, A. T., Camprodon, J. A., Pascual-Leone, A. & Goebel, R. The dynamics of interhemispheric compensatory processes in mental imagery. *Science* 308, 702-704 (2005).

3 Cohen Kadosh, R. *et al.* Virtual dyscalculia induced by parietal-lobe TMS impairs automatic magnitude processing. *Cur Biol* 17, 689-693 (2007).

4 Sack, A. T., Kohler, A., Linden, D. E. J., Goebel, R. & Muckli, L. The temporal characteristics of motion processing in hMT/V5+: Combining fMRI and neuronavigated TMS. *NeuroImage* 29, 1326-1335 (2006).

5 Lunney, G. H. Using analysis of variance with a dichotomous dependent variable: an empirical study. *J Educ Meas* 7, 263-269 (1970).

6 Glass, G. V., Peckham, P. D. & Sanders, J. R. Consequences of failure to meet assumptions underlying the fixed effects analyses of variance and covariance. *Rev Educ Res* 42, 237-288 (1972).

7 Pachella, R. in *Human information processing: Tutorials in performance and cognition* (ed B. H. Kantowitz) 41–82 (Erlbaum, 1974).

8 JASP v. 0.8.0.0 (2016).

9 Snowball, A. *et al.* Long-Term Enhancement of Brain Function and Cognition Using Cognitive Training and Brain Stimulation. *Cur. Biol.* 23, 987-992, doi:10.1016/j.cub.2013.04.045 (2013).

10 Cappelletti, M. *et al.* Transfer of cognitive training across magnitude dimensions achieved with concurrent brain stimulation of the parietal lobe. *J. Neurosci.* 33, 14899-14907 (2013).

11 Popescu, T. *et al.* Transcranial random noise stimulation mitigates increased difficulty in an arithmetic learning task. *Neuropsychologia* 81, 255-264 (2016).

12 Fertonani, A., Pirulli, C. & Miniussi, C. Random noise stimulation improves neuroplasticity in perceptual learning. *J. Neurosci.* 31, 15416-15423 (2011).
